# Blood lead level and its association with body mass index and obesity in China - Results from SPECT-China study

**DOI:** 10.1038/srep18299

**Published:** 2015-12-14

**Authors:** Ningjian Wang, Chi Chen, Xiaomin Nie, Bing Han, Qin Li, Yi Chen, Chunfang Zhu, Yingchao Chen, Fangzhen Xia, Zhen Cang, Meng Lu, Ying Meng, Hualing Zhai, Dongping Lin, Shiyong Cui, Michael D. Jensen, Yingli Lu

**Affiliations:** 1 Institute and Department of Endocrinology and Metabolism, Shanghai Ninth People’s Hospital, Shanghai JiaoTong University School of Medicine, Shanghai, China; 2 The Center for Disease Prevention and Control of Baoshan District, Shanghai, China; 3 Endocrine Research Unit, Mayo Clinic, Rochester, MN, USA

## Abstract

We aimed to report environmental and blood lead level (BLL) in China, and investigate the relationship of BLL with body mass index (BMI) and obesity. 5558 subjects were enrolled from 16 sites in China. BLL was measured by atomic absorption spectrometry. Obesity was defined as BMI ≥ 30 kg/m^2^. Median (interquartile range) of BLL was 44.00 μg/L (29.00–62.16) for men and 37.79 μg/L (25.13–54.35) for women, about twice higher than in U.S. population. Subjects in rural and high-economic-status areas had significantly greater BLL (*P* < 0.001). However, in these areas, the lead levels in drinking water, river water and rice were comparable to or significantly lower than those in urban and low-economic-status areas. After adjustment for age, urbanization, economic status and metabolic factors, BLL was independently associated with BMI in women (*P* for trend < 0.001), but not in men. In fully adjusted model, increased quartiles of BLL were associated with significantly increased odds ratios of obesity (*P* for trend < 0.01) in women. In conclusion, BLLs in Chinese adults were much higher than in developed countries. There was a sex-specific association between BLL and BMI. Elevated BLL does not appear to be associated with lead levels in drinking water or rice, suggesting some other exposure source.

Between 1992 and 2012, the prevalence of overweight and obesity increased in all gender and age groups and in all geographic areas. Based on national survey data, the combined prevalence of overweight and obesity rose from 14.6%^1^ to 32.3%^2^. The rise in prevalence of obesity coincides with increased exposure to environmental toxins, particularly those so-called endocrine disrupting chemicals^3^.

Lead is highly toxic and carcinogenic for humans^4^, especially damaging the nervous system and causing brain disorders. For decades, leaded gasoline was the primary source of human exposure to lead^5^. Although leaded gasoline in China was banned in 2000, lead-acid battery industry in China may become another big source of blood lead. China’s lead-acid battery industry is the world’s largest in terms of production and consumption, which uses over 67% of China’s total lead production^5^. In the process of manufacturing, lead exhaust may be released as air pollution or waste mixtures into soil and waterways^5^. Drinking water pipe systems is also one sources of lead in the environment^6^. From air, soil, water and food, lead could be up taken mainly through ingestion and inhalation^7^. In population aged 0–18 years old of China, about half of them had blood lead levels (BLL) higher than 45 μg/L^8^, when chelation could be considered^9^. However few studies have reported BLL in Chinese adults with a large sample.

Lead is considered to be one of endocrine disrupting chemicals, which may be associated with hypothalamic-pituitary-adrenal axis function, thyroid hormones and bone metabolism^10^,^11^,^12^,^13^. It may also associate with obesity. In animal studies, lead exposure was associated with a trend of increased wean bodyweight^7^ and even low-level human equivalent gestational lead exposure produces late-onset obesity^14^. Epidemiologic studies exploring the association between BLL and obesity are inconclusive. Kim *et al.* reported positive association between lead levels in dentin and body mass index (BMI)^15^, whereas from data of National Health and Nutrition Examination Survey (NHANES) 1999–2006 in USA, BLLs were associated with lower BMI in adults^3^. There are also other studies that did not find an association between BMI and lead exposure in adults^16^,^17^.

There has been no study exploring the association of BLL and obesity in Chinese adults. The 2014 **S**urvey on **P**revalence in **E**ast **C**hina for Me**t**abolic Diseases and Risk Factors (SPECT-China, 2014) measured BLL in a Chinese population. We hypothesized that BLL might be associated with body mass index and obesity. Using these data, we aimed to investigate the current environmental lead status in China and BLL, and further explore the association between BLL and obesity.

## Results

### Characteristics of participants

General characteristics of the participants are summarized in Table 1. Prevalence of overweight and obesity were 36.7% and 5.1% in men, and 27.9% and 5.6% in women. Medians (IQR) of BLL were 44.00 μg/L (29.00–62.16) for men and 37.79 μg/L (25.13–54.35) for women. Compared with the participants with normal BMI, those with overweight and obesity had higher levels of BLL in women (40.30 (26.00–58.74) and 44.35 (28.47–63.68) vs. 36.35 (25.00–52.00), *P* < 0.05), but not in men.

Table 2 shows characteristics of study population according to BLL quartiles. With the increasing BLL quartiles, women had significantly greater BMI, LDL and triglycerides (*P* for trend < 0.01) and also higher prevalence of obesity, diabetes and hypertension (*P* for trend < 0.01). No significant trend for BMI was observed across the BLL quartiles in men (*P* for trend 0.171).

### Association of BLL with BMI by linear regression

**Figure 1:**
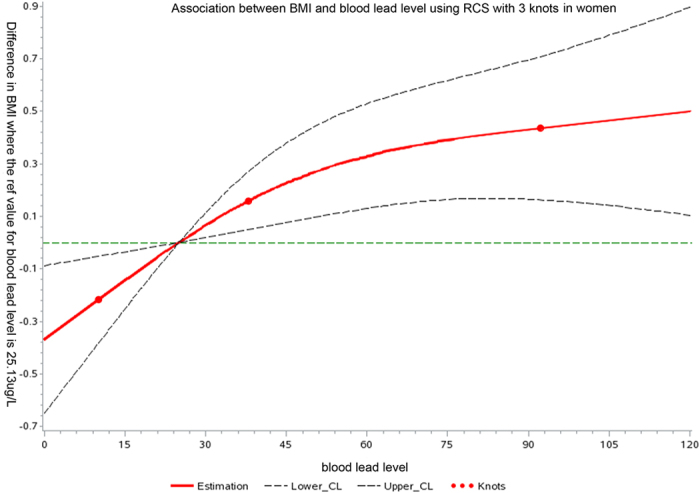
Dose–response association between BMI and BLL in women adjusted for age, rural/urban residence, economic status, current smoking, diabetes, dyslipidemia and hypertension. A solid line shows dose–response curve between BLL and BMI. Y-axis represents the difference in BMI between individuals with any value of BLL with individuals with 25.13 μg/L of BLL. The dashed lines represent the 95% confidence of interval. Knots are represented by dots.

Table 3 reports the results of the linear regression models exploring the association of BLL with BMI. Using the lowest quartile of BLL as the reference, significant positive crude (model 1) trend was observed for BLL with BMI in women (*P* for trend < 0.001), but not in men. In the fully adjusted model (model 3), after adjustment for age, rural/urban residence, economic status, current smoking, diabetes, dyslipidemia and hypertension, BLL remained significantly positively associated with BMI in women (*P* for trend < 0.001). No significant trend was observed in men (*P* for trend 0.82). The dose-relationship between BLL and BMI was further confirmed by restricted cubic spline analyses in women (Fig. 1) and men (Supplementary Fig. S1).

### Association of BLL with overweight and obesity

Table 4 demonstrates the results of the multinomial logistic regression measuring the association of BLL with overweight and obesity. In unadjusted model, compared with women in the lowest quartile of BLL, ORs of overweight and obesity in women in the highest quartile were 1.36 (95% CI 1.10, 1.69) and 2.09 (95% CI 1.36, 3.21). After adjustment for demographic variables, diabetes, dyslipidemia and hypertension, OR in overweight showed no significance and OR in obese subjects was slightly attenuated but still showed significance (OR 1.86, 95% CI 1.16, 2.96, *P* for trend < 0.01). The probability of overweight and obesity did not significantly increase or decrease in all models in men.

### Lead levels by economic status and urbanization

Supplementary Table S1 and S2 summarize the results of blood and environmental lead levels and metabolic factors by economic status and urbanization. Compared with subjects living in areas with low economic status, subjects in areas with high economic status had significantly greater BLL (*P* < 0.001), and these women also had greater BMI and higher prevalence of obesity (*P* < 0.001). In areas with high economic status, the lead level in drinking water was comparable to that in areas with low economic status but lead level in river water and rice was significantly lower (*P* < 0.05).

Meanwhile, subjects in rural areas had significantly greater BLL (*P* < 0.001) and women in rural areas had greater BMI as well as higher prevalence of obesity (*P* < 0.01). Rural areas had comparable lead level in drinking water to urban areas but they had lower lead concentrations in rice and river water (P < 0.05).

Supplementary Table S3 also summarizes environmental and blood lead level by the territorial origin. In women, subjects in rural areas in Shanghai (high economic status) had significantly higher BLL and BMI than in urban areas in Jiangxi Province (low economic status) (*P* < 0.05).

## Discussion

**Figure 2:**
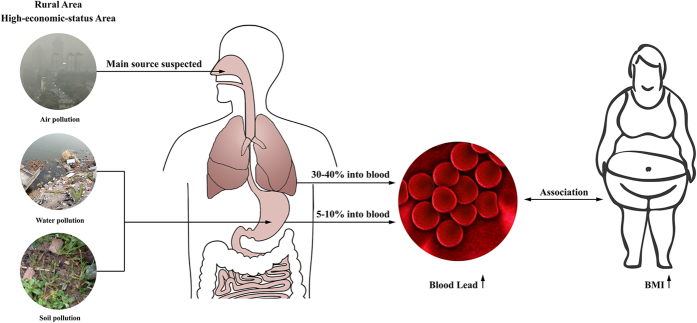
The environment and blood lead status and its association with obesity in China. Based on the largest sample of general women ever reporting blood lead level (BLL) in China, BLL in Chinese women was about twice higher than in U.S. population. Women in rural and rapid-economic-development area had higher BLL. Blood lead is mainly from air, water, soil and food. In our study, elevated BLL does not appear to be associated with lead levels in drinking water or rice, suggesting some other exposure source. Moreover, we found that BLL was positively associated with BMI and obesity after adjustment for age, residence area, economic status, diabetes, dyslipidemia and hypertension in Chinese women. This figure was created by Adobe Illustrator CS5 and Adobe Photoshop CS5 (Adobe Systems Incorporated, USA).

As far as we know, this study reported BLL in the largest sample of general Chinese population and it provided newest data about BLL as well as environmental lead in East China. This is also the first study to explore the association between BLL and BMI in Chinese adults. We found that BLL was positively associated with BMI and obesity after adjustment for age, residence area, economic status, smoking, diabetes, dyslipidemia and hypertension in Chinese women, but not in men. Also, elevated BLL does not appear to be associated with lead levels in drinking water or rice, suggesting some other exposure source (Fig. 2).

The most recent BLL data in general Chinese population we could find were from just one local area about 5 years ago^18^. Meanwhile the samples of almost all previous reports were relatively small, from dozens to hundreds^19^,^20^. Though the BLL has gradually dropped in the past decades^8^,^19^,^20^, the medians of BLL in Chinese were still about twice higher than in Americans (NHANES, 2003–2006)^3^. Most of BLLs in Chinese subjects were in the normal range according to the Centers for Disease Control and Prevention of USA (<100 ug/L)^21^. However, epidemiological results exploring BLL and mortality imply there may be no safe threshold BLL^22^. Concerted efforts are still warranted to reduce adult lead exposure.

We found subjects in rural areas and under high economic status had greater BLL. Since China’s reform and opening up in 1978, many factories had been set up or moved into the rural or suburban areas^23^. It has brought improved urbanization and economic development in these areas, but it may also bring more sever environmental pollution and metabolic disorders such as obesity, diabetes, dysglycemia and hypertension. And we detected independent association of BLL with BMI and obesity in women. Hence, rural area, especially with high economic status should be one of the critical prevention targets for metabolic diseases in China and lead pollution should be managed.

Elevated BLL does not appear to be associated with lead levels in drinking water, river water or rice, suggesting the existence of some other exposure source. For example, subjects under high economic status had greater BLL than those under low economic status; however, lead levels in drinking water, river water, and rice were similar or higher within areas reported to have low economic status. Blood lead is mainly from air, water, soil and food^6^. In adults about 30% to 40% of inhaled but only 5% to 10% of ingested lead is absorbed into blood^6^. Thus, we suspected the one of main sources of BLL might be air pollution in East China, which need further investigation (Fig. 2).

Limited studies explored the association between BLL and obesity in adults. In 2013, Franco Scinicariello *et al.* reported that BLL associated with lower body weight based on data from NHANES 1999–2006^3^. Adults in the highest BLL quartile were less likely to be obese (OR = 0.42, 95% CI: 0.35–0.50) compared to those in the lowest BLL quartile. However we found that BLL positively associated with BMI in women. In the study of Franco Scinicariello *et al.* most of their subjects were white, black and Mexican-American, so the data might not be generalizable to other ethnic groups. In another study in Asian population, a higher prevalence of MS is associated with higher blood lead levels, which may partly support the ethnicity difference^24^. Furthermore, BLLs in our subjects were much higher than in the Americans. When U.S. population’s BLL was as high as 50–90 μg/L about three decades ago, this BLL level was associated with an increased risk of death from all causes, cardiovascular disease, and cancer^4^. Finally, they did not perform sex-specific analyses, though sex was adjusted. Thus, we suspect different BLLs may have different effects on human health, which warrants further investigation.

The results of association between lead exposure and BMI are far from being conclusive. In another longitudinal study in Boston, the chronic lead exposure in childhood may result in obesity that persists into adulthood^15^. In 1975–1978, among 236 children, a 10-fold increase in dentin lead level was associated with an increase of 1.02 kg/m^2^ in BMI. 58 of these children were reexamined in 1989–1990 when their ages were from age 7 to age 20. A 10-fold increase in dentin lead level was also associated with an increase in BMI change of 2.65 kg/m^2 15^. In another two epidemiological studies, No association was found between BLL and BMI or obesity in adults^16^,^17^.

There are several studies supporting that animals exposed to lead experience obesity^7^,^14^,^25^. Christopher Faulk *et al.* demonstrated perinatal lead exposure at BLL between 4.1 μg/dL and 32 μg/dL was associated with increased food intake, body weight and total body fat in male mice, and lead acted in a locus-specific fashion, potentially dependent on the genomic feature hosting the CpG site of interest^7^,^25^. J. Leigh Leasure *et al.* also reported that increases in body weight were observed in year-old gestational lead exposure male mice^14^.

Why did BLL have sex-specific association with obesity in our study? First, a study investigated the association between prenatal exposure to lead and the risk of low birth weight. The association was significant among female infants, but not in male infants^26^. As we know, low birth weight is a risk factor for higher BMI and obesity in adulthood^27^. This prolonged effect may not be excluded in adult female today. Second, sex hormones may be involved. It is well known that sex hormones are closely related to the regulation of adiposity in men and women^28^. Some studies also explored the association between BLL and sex hormones, though they are limited and inconclusive. John Meeker *et al.* reported in men BLL was not associated with total and free testosterone, follicle-stimulating hormone and luteinizing hormone^29^. However, in women it was reported BLL is related to and a significant predictor of serum estradiol^30^. Knowledge on this sex-specific association is still very limited which needs further investigation.

Several candidates may explain the lead-induced obesity. First, lead has been linked to altered hypothalamic-pituitary-adrenal axis^31^,^32^, which can induce obesity^33^. Even low BLL can alter children’s adrenocortical responses to acute stress. Gump BB *et al.* reported lead exposure was not associated with initial salivary cortisol levels but after an acute stressor, high BLL was significantly associated with higher cortisol responses, indicating a lead-induced hypothalamic-pituitary-adrenal axis dysregulation^34^. Actually, lead exposure itself could also induce a stress-like response and elevate ACTH and corticosterone concentrations^31^. Second, there is clear evidence that oxidative stress and fat metabolism abnormality could form a vicious circle and lead to obesity^35^. Studies showed that lead induced the generation of reactive oxygen species while inhibiting their scavenging and neutralization by antioxidant defense mechanisms^36^,^37^, so it is reasonable to deduce that lead exposure may induce obesity partly through oxidative stress^24^.

Our study had some strengths. First, the significance, we provided newest data about BLL in China, which may be important for Chinese public healthcare. It is also the first study to detect the association between BLL and obesity in Chinese. Second, this study has strong quality control, because the same trained research group completed all anthropometric measurements and questionnaires. Third, our study was community dwelling population-based design, with a large sample size, so our results are more reflective as opposed to a clinic-based population.

However, our study also has some limitations. First, due to the cross-sectional study nature, we cannot draw causal relationship between BLL and obesity. Second, Han Chinese were primarily recruited and the data may not be generalizable to other ethnic groups. Finally, we have some confounders adjusted, but this study did not include some covariates such as working in metal industry or not, outdoor physical activity, overall time spent outdoor. Take outdoor physical activity for example: because one major source of BLL is air-borne lead^19^, individuals with longer outdoor physical activity might take more lead through inhalation but have less risk for weight gain^38^. This point may lead to some potential bias of the study findings. More studies are needed to confirm this association by investigating the underlying mechanism and adjusting more potential covariates.

In conclusion, BLLs in Chinese were still much higher than in developed countries. BLL was positively associated with BMI and obesity in Chinese women, but not in men. Concerted efforts are warranted to reduce adult lead exposure.

## Methods

### Study population

**Figure 3:**
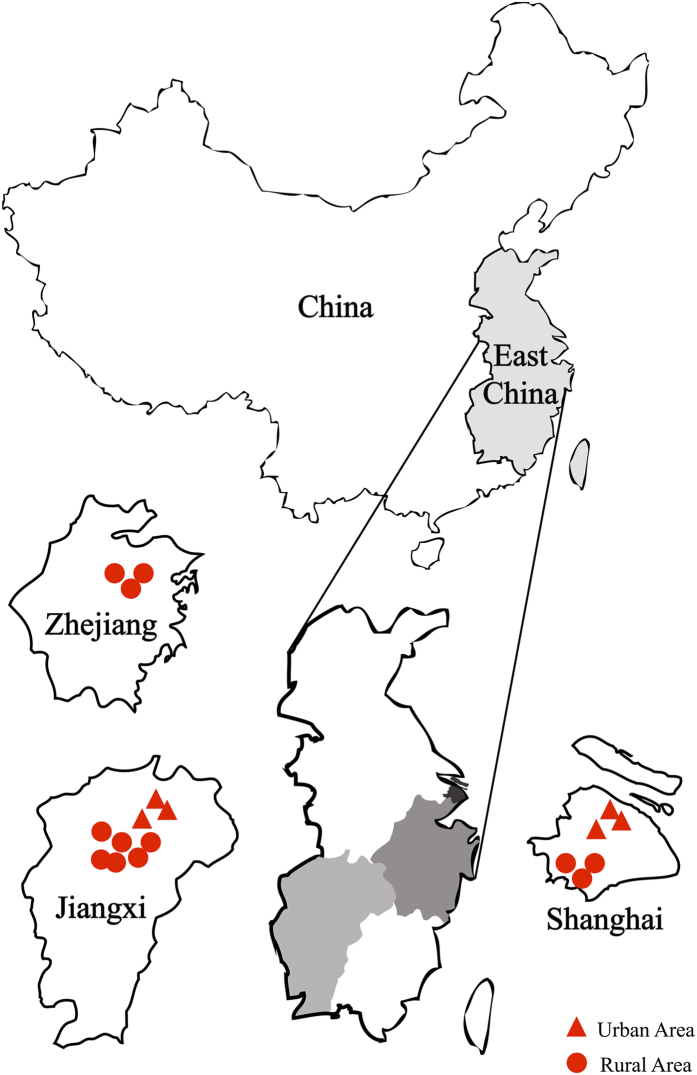
Locations of the survey sites in East China. This study was performed in three sites in urban areas of Shanghai, one site (three spots) in an urban area of Jiangxi, three sites in rural areas in Shanghai, three sites in rural areas in Zhejiang and six sites in rural areas in Jiangxi. This figure was created by Adobe Illustrator CS5 (Adobe Systems Incorporated, USA).

**Figure 4:**
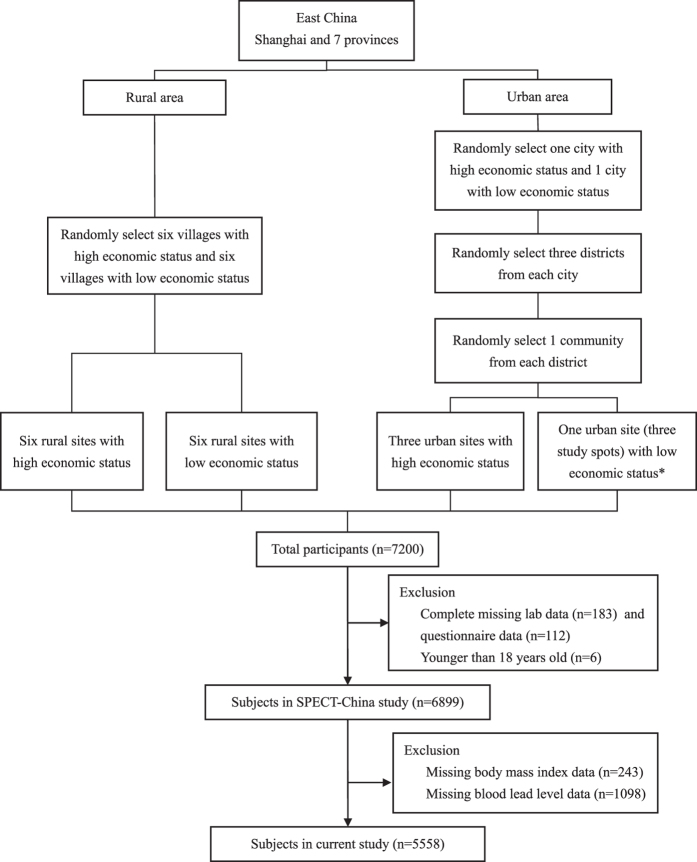
Flowchart of sampling frame and participants selected from SPECT-China. *Because the 3 communities chosen were all very large communities, to maintain the study feasibility and avoid oversampling in this area, we randomly chose 1 community (3 study spots) from these 3 communities.

SPECT-China is a population-based cross-sectional survey on prevalence of metabolic diseases and risk factors in East China from February to June 2014, where 99.5% of residents are Han Chinese. Registration number is ChiCTR-ECS-14005052 (www.chictr.org). The study protocol was approved by the Ethics Committee of Shanghai Ninth People’s Hospital, Shanghai JiaoTong University School of Medicine. All procedures followed were in accordance with the ethical standards of the responsible committee on human experimentation (institutional and national) and with the Helsinki Declaration of 1975, as revised in 2008. All participants provided written informed consent before data collection. A stratified cluster sampling method was used, as previously reported^39^. The first sampling level was by rural and urban residence and the second sampling level was by area economic status. In urban areas, we randomly chose 1 city with a low economic status and 1 city with a high economic status. Then, in both cities, we randomly chose 3 districts from which 3 communities were randomly selected. Because the 3 communities chosen in Jiangxi Province were all very large communities, to maintain the study feasibility and to avoid oversampling in this area, we randomly chose 1 community (3 study spots) from these 3 communities. In rural areas we randomly chose 6 villages with low economic status and 6 villages with high economic status. From February to June 2014, this study was performed in 3 urban sites in Shanghai, 1 urban site in Jiangxi Province, 3 rural sites in Shanghai, 3 rural sites in Zhejiang and 6 rural sites in Jiangxi Province (Fig. 3). Inclusion criteria were Chinese citizens aged 18 years old and above. Those with severe communication problems and acute illness and who were pregnant women and unwilling to participate were excluded. 7,200 people participated in this investigation. We excluded participants who were missing lab results (n = 183), were missing questionnaire data (n = 112), and were younger than 18 years old (n = 6). 6899 subjects were enrolled in SPECT-China study^39^. Then, participants with missing values of BMI (n = 243) and BLL (n = 1098) were excluded. Finally, this study included a total number of 5558 subjects with a mean ± SD age of 53 ± 13 years. (Fig. 4).

### Measurements

In every site, the same trained staff completed the questionnaire including information on demographic characteristics, medical history and lifestyle risk factors, and collected anthropometric data. Current smoking was defined as having smoked at least 100 cigarettes in one’s lifetime and currently smoking cigarettes^40^. Body weight, height, and blood pressure were measured with standard methods as described previously^40^. BMI was calculated as weight in kilograms divided by height in meters squared.

Venous blood samples were drawn after an overnight fast of at least 8h. The blood samples for plasma glucose test were centrifuged on the spot in 1 hour after collection. Blood samples were stored at −20 °C and shipped by air to a central laboratory within 2–4 hours of collection. The central laboratory is certified by the College of American Pathologists. BLL was determined by atomic absorption spectrometry (BH2200, China). Glycated hemoglobin (HbA1c) was assessed by high-performance liquid chromatography (MQ-2000PT, China). Fasting plasma glucose (FPG), total cholesterol, triglycerides (TG), high-density lipoprotein (HDL) and high-density lipoprotein (LDL) were measured by BECKMAN COULTER AU 680 (Germany). Insulin was detected by chemiluminescence (Abbott i2000 SR, USA).

### Environmental sample collection and analysis

In the studied areas, the drinking water is from tap, well and water fountain. A total of 140 drinking water samples (28 from rural sites in Zhejiang, 58 from rural sites in Shanghai, 6 from rural sites in Jiangxi, 15 from urban sites in Jiangxi, and 33 from rural sites in Shanghai) were collected. We also collected 37 river water samples (13 from rural sites in Zhejiang, 15 from rural sites in Shanghai, 4 from rural sites in Jiangxi and 5 from urban sites in Jiangxi). The water samples were collected in 0.5 L in pre-conditioned acid-washed polyethylene bottles from participants’ families or from rivers in different geographic sites. They were stored at −20 °C until analysis. The analysis was based on national standard examination methods [GB/T5750.6-2006(11.1)]. The concentrations of lead in water were measured by flameless atomic absorption spectrometry (atomic absorption spectrometer, Thermo Fischer Scientific, USA). Standard reference material [GBW(E)080278] obtained from the Shanghai Institute of Measurement and Testing, China was used for validation of the analytical procedure.

Rice was sampled because it is eaten in high amounts in East China and about 45% of dietary intake of lead was from cereal^41^. A total of 92 rice samples were collected (30 from rural sites in Zhejiang, 30 from rural sites in Shanghai, 10 from rural sites in Jiangxi, 10 from urban sites in Jiangxi, and 12 from rural sites in Shanghai). All rice samples were collected directly from participants’ homes. The analysis was based on national standard examination methods [GB/T5009.12-2010]. The concentrations of lead in rice were measured by graphite furnace atomic absorption spectrometry (atomic absorption spectrometer, Thermo Fischer Scientific, USA).

### Definition of variables and outcomes

Overweight and obesity were defined based upon BMI measures of 25–29.9 kg/m^2^ and ≥30 kg/m^2^, respectively^40^. Based on American Diabetes Association 2014 criteria, diabetes was defined as a previous diagnosis by healthcare professionals, FPG ≥ 7.0 mmol/L, or HbA1c ≥ 6.5%. Hypertension was assessed by systolic blood pressure ≥140 mmHg, diastolic blood pressure ≥90 mmHg or self-reported previous diagnosis of hypertension by physicians. Dyslipidemia was defined according to the modified National Cholesterol Education Program-Adult Treatment Panel III as total cholesterol ≥6.22 mmol/L (240 mg/dL), triglycerides ≥2.26 mmol/L (200 mg/dL), LDL-C ≥ 4.14 mmol/L (160 mg/dL), HDL-C < 1.04 mmol/L (40 mg/dL), or self-reported previous diagnosis of hyperlipidemia by physicians^42^.

We took residence area as a covariate, since in China the prevalence of obesity in rural and urban area is different^43^. Economic development status was measured by the mean GDP per capita of whole nation (6807 US dollars from World Bank) in 2013 as the cutoff point for each site. If the GDP per capita of the site was higher than the national GDP per capita, it was regarded as having a high economic status, and vice versa.

### Statistical Analysis

Analyses were performed with IBM SPSS Statistics, version 22 (IBM Corporation, Armonk, NY, USA). All analyses were two-sided. A *P* value < 0.05 indicated a significant difference. General characteristics were summarized as median (interquartile range) for continuous variables or as number with proportion for categorical variables. Kolmogorov-Smirnov test and P-P plots were used to determine whether the data were normally distributed. To test for differences of variables between different groups, Mann-Whitney U or Kruskal-Wallis test was used for continuous variables with skewed distribution, and Pearson chi-square test for categorical variables.

Age, economic status, rural/urban residence, current smoking, blood cadmium level, diabetes, dyslipidemia and hypertension were examined as confounders or independent predictors of the association between BLL and BMI. Economic status and rural/urban residence have been associated with body weight and blood lead level^8^,^43^. Smoking still is a substantial source of exposure to lead in general population^44^ and associated with weight change^45^. Diabetes, dyslipidemia and hypertension were well-known comorbidities or risk factors of weight gain which were adjusted further.

The association of BLL quartiles (independent variable) with BMI (dependent variables) was assessed by linear regression analysis. Results were expressed as unstandardized coefficients (standard error). Model 1 was unadjusted. Model 2 was adjusted for potential confounders including age, rural/urban residence, economic status and smoking. Model 3 was further adjusted for comorbidities or potential mediators including diabetes, dyslipidemia and hypertension.

To further determine the relationship between BLL and BMI, we used BLL as restricted cubic spline. The SAS macro-written Desquilbet and Mariotti^46^ was used to perform restricted cubic spline analyses. The knots were 10th, 50th, and 90th percentile as recommended by previous studies^3^.

To consider overweight and obesity probability model, the multinomial logistic regression analysis was applied with the lowest BLL quartile as reference. Data were expressed as odds ratio (OR) (95% CI). The models were the same as those in linear regression analysis. Interaction effect was tested between BLL and diabetes, dyslipidemia and hypertension by adding a multiplicative factor in the logistic regression model.

## Additional Information

**How to cite this article**: Wang, N. *et al.* Blood lead level and its association with body mass index and obesity in China - Results from SPECT-China study. *Sci. Rep.* **5**, 18299; doi: 10.1038/srep18299 (2015).

## Supplementary Material

Supplementary Information

## Figures and Tables

**Table 1:** Characteristics of participants categorized by body mass index.

	Normal	Overweight	Obesity
Men			
* N* (%)	1301 (58.2)	820 (36.7)	114 (5.1)
* *Age, yr	54 (43–64)	53 (43–62)	55 (47–63)
* *HDL, mmol/L	1.41 (1.21–1.61)	1.23 (1.09–1.43)*	1.23 (1.03–1.39)*
* *LDL, mmol/L	2.72 (2.31–3.18)	3.07 (2.65–3.51)*	3.12 (2.57–3.66)*
* *Blood lead level, μg/L	44.90 (30.00–63.51)	43.02 (28.80–61.00)	43.75 (26.70–60.12)
* *Triglycerides, mmol/L	1.20 (0.90–1.72)	1.79 (1.26–2.57)*	2.09 (1.49–2.97)*^†^
* *Diabetes, %	10.9	16.8*	26.3*^†^
* *Dyslipidemia, %	23.8	47.0*	60.5*^†^
* *Hypertension, %	39.1	53.6*	72.6*^†^
* *Current smoker, %	48.0	47.4	57.9^†^
* *Rural/urban residence, %	58.2/41.8	47.1/52.9*	42.1/57.9*
* *Low/high economic status, %	25.6/74.4	30.4/69.6*	29.8/70.2
Women			
* N* (%)	2209 (66.5)	928 (27.9)	186 (5.6)
* *Age, yr	51 (40–61)	57 (48–63)*	58 (50–65)*
* *HDL, mmol/L	1.53 (1.34–1.76)	1.42 (1.24–1.64)*	1.36 (1.18–1.58)*^†^
* *LDL, mmol/L	2.76 (2.33–3.25)	3.01 (2.59–3.54)*	3.10 (2.70–3.50)*
* *Blood lead level, μg/L	36.35 (25.00–52.00)	40.30 (26.00–58.74)*	44.35 (28.47–63.68)*
* *Triglycerides, mmol/L	1.08 (0.81–1.51)	1.45 (1.06–2.03)*	1.65 (1.22–2.29)*^†^
* *Diabetes, %	6.8	15.2*	21.0*
* *Dyslipidemia, %	22.7	38.4*	48.6*
* *Hypertension, %	30.5	50.9*	66.5*^†^
* *Current smoker, %	2.4	2.2	4.1
* *Rural/urban residence, %	55.5/44.5	60.5/39.5*	67.2/32.8*
* *Low/high economic status, %	32.1/67.9	25.9/74.1*	23.7/76.3*

HDL, high-density lipoprotein; LDL, low-density lipoprotein.

*P < 0.05, compared with normal group; ^†^P < 0.05, compared with overweight group.

Data are summarized as median (interquartile range) for continuous variables or as number with proportion for categorical variables. Kruskal-Wallis test were used for continuous variables and Pearson chi-square test for categorical variables.

Overweight and obese adults were defined based upon BMI measures of 25–29.9 kg/m^2^ and ≥30 kg/m^2^, respectively. Dyslipidemia was defined as total cholesterol ≥6.22 mmol/L, triglycerides ≥2.26 mmol/L, LDL-C ≥ 4.14 mmol/L or HDL-C < 1.04 mmol/L, or self-reported previous diagnosis of hyperlipidemia by physicians.

**Table 2:** Characteristics of the study population by blood lead level quartiles.

	Quartile 1	Quartile 2	Quartile 3	Quartile 4	*P* for trend
Men					
* N*	561	560	556	558	
* *Blood lead level, μg/L	≤29.00	29.01–44.00	44.01–62.16	≥62.17	
* *Age, yr	52 (39–61)	52 (42–61)	55 (44–63)	58 (47–65)	<0.001
* *HDL, mmol/L	1.31(1.12–1.51)	1.32 (1.13–1.54)	1.32 (1.13–1.57)	1.37 (1.17–1.61)	<0.001
* *LDL, mmol/L	2.91 (2.46–3.39)	2.82 (2.38–3.28)	2.86 (2.43–3.35)	2.93 (2.45–3.35)	0.624
* *Body mass index, kg/m^2^	24.5 (22.3–26.6)	24.4 (22.0–26.6)	24.3 (21.9–26.6)	23.9 (21.9–26.6)	0.171
* *Triglycerides, mmol/L	1.43 (0.99–2.17)	1.43 (1.02–2.08)	1.42 (1.00–2.11)	1.37 (1.01–1.97)	0.575
Weight, %					
* *Overweight	38.1	37.5	37.1	34.1	0.132
* *Obesity	5.7	4.5	5.6	4.7	0.456
Diabetes, %	14.6	12.0	13.8	15.1	0.618
Dyslipidemia, %	36.2	34.8	36.0	29.7	<0.05
Hypertension, %	41.0	42.1	49.5	52.2	<0.001
Current smoker, %	43.8	43.8	50.4	55.2	<0.001
Rural/urban residence, %	36.0/64.0	43.6/56.4	61.3/38.7	72.4/27.6	<0.001
Low/high economic status, %	31.2/68.8	35.0/65.0	26.3/73.7	17.7/82.3	<0.001
Women					
* N*	831	831	831	830	
* *Blood lead level, μg/L	≤25.13	25.14–37.79	37.80–54.35	≥54.36	
* *Age, yr	51 (40–60)	51 (41–61)	53 (43–62)	57 (47–65)	<0.001
* *HDL, mmol/L	1.48 (1.29–1.68)	1.51 (1.31–1.72)	1.50 (1.31–1.71)	1.50 (1.30–1.76)	<0.05
* *LDL, mmol/L	2.81 (2.35–3.33)	2.80 (2.36–3.27)	2.88 (2.41–3.38)	2.94 (2.50–3.37)	<0.01
* *Body mass index, kg/m^2^	23.3 (21.1–25.7)	23.1 (21.1–25.5)	23.7 (21.5–25.9)	24.1 (21.6–26.7)	<0.001
* *Triglycerides, mmol/L	1.15 (0.83–1.68)	1.17 (0.86–1.63)	1.24 (0.89–1.76)	1.23 (0.94–1.75)	<0.01
Weight, %					
* *Overweight	26.7	23.8	29.4	31.8	<0.001
* *Obesity	4.2	5.1	5.4	7.7	<0.001
Diabetes, %	8.2	9.4	8.9	13.3	<0.01
Dyslipidemia, %	22.7	21.8	24.9	24.6	0.190
Hypertension, %	32.3	33.7	39.6	43.7	<0.001
Current smoker, %	3.1	2.2	2.0	2.3	0.278
Rural/urban residence, %	46.2/53.8	49.7/50.3	58.4/41.6	76.0/24.0	<0.001
Low/high economic status, %	33.5/66.5	35.9/64.1	30.8/69.2	19.3/60.7	<0.001

HDL, high-density lipoprotein; LDL, low-density lipoprotein.

Data are summarized as median (interquartile range) for continuous variables, or as number with proportion for categorical variables.

Overweight and obese adults were defined based upon BMI measures of 25–29.9 kg/m^2^ and ≥30 kg/m^2^, respectively. Dyslipidemia was defined as total cholesterol ≥6.22 mmol/L, triglycerides ≥2.26 mmol/L, LDL-C ≥ 4.14 mmol/L or HDL-C < 1.04 mmol/L, or self-reported previous diagnosis of hyperlipidemia by physicians.

**Table 3:** Association of blood lead level quartiles (independent variable) with body mass index (dependent variable).

Body mass index	Blood lead level, ug/dl				*P* for trend
	Q1	Q2	Q3	Q4	
Men					
Model 1 (SE)	Ref.	−0.19 (0.20)	−0.11 (0.20)	−0.31 (0.20)	0.19
Model 2 (SE)	Ref.	−0.11 (0.20)	0.13 (0.21)	0.002 (0.21)	0.73
Model 3 (SE)	Ref.	−0.06 (0.19)	0.05 (0.19)	0.01 (0.20)	0.82
Women					
Model 1 (SE)	Ref.	−0.11 (0.18)	0.43 (0.18)*	0.89 (0.18)*	<0.001
Model 2 (SE)	Ref.	−0.12 (0.17)	0.30 (0.17)	0.59 (0.18)*	<0.001
Model 3 (SE)	Ref.	−0.12 (0.17)	0.27 (0.17)	0.59 (0.17)*	<0.001

Data are expressed as unstandardized coefficients (standard errors). Linear regression analyses were used. Model 1 was unadjusted. Model 2 included terms for age, rural/urban residence, economic status and current smoking. Model 3 included terms for model 2, diabetes, dyslipidemia and hypertension.

*Denotes statistical significance at *P* < 0.05.

**Table 4:** Association of blood lead level with overweight and obesity.

Body mass index	Blood lead level, ug/dl				*P* for trend
	Q1	Q2	Q3	Q4	
Men					
* *Overweight					
* *Model 1	1.00 (Ref)	0.95(0.74, 1.22)	0.95(0.74, 1.22)	0.82(0.64, 1.05)	0.13
* *Model 2	1.00 (Ref)	1.00(0.78, 1.29)	1.04(0.81, 1.35)	0.95(0.73, 1.24)	0.82
* *Model 3	1.00 (Ref)	1.02(0.79,1.33)	1.01(0.77, 1.32)	0.95(0.72,1.26)	0.74
* *Obesity					
* *Model 1	1.00 (Ref)	0.76(0.44, 1.31)	0.96(0.57, 1.61)	0.75(0.44, 1.28)	0.46
* *Model 2	1.00 (Ref)	0.75(0.42, 1.34)	1.19(0.69, 2.04)	0.90(0.50, 1.61)	0.92
* *Model 3	1.00 (Ref)	0.73(0.40, 1.33)	1.13(0.64, 1.97)	0.88(0.48, 1.61)	0.99
Women					
* *Overweight					
* *Model 1	1.00 (Ref)	0.87(0.69, 1.08)	1.16(0.94, 1.45)	1.36(1.10, 1.69)	<0.001
* *Model 2	1.00 (Ref)	0.83(0.66, 1.04)	1.08(0.87, 1.36)	1.14(0.91, 1.43)	0.08
* *Model 3	1.00 (Ref)	0.82(0.65, 1.04)	1.07(0.85, 1.35)	1.16(0.92, 1.46)	0.07
Obesity					
* *Model 1	1.00 (Ref)	1.17(0.73, 1.85)	1.36(0.86, 2.15)	2.09(1.36, 3.21)	<0.001
* *Model 2	1.00 (Ref)	1.20(0.74, 1.96)	1.35(0.84, 2.20)	1.83(1.15, 2.92)	<0.01
* *Model 3	1.00 (Ref)	1.16(0.70, 1.90)	1.31(0.80, 2.14)	1.86(1.16, 2.98)	<0.01

Data are odds ratio (95% confidence interval). Multinomial logistic regression analyses were performed. Model 1 was unadjusted. Model 2 included terms for age, rural/urban residence, economic status and smoking. Model 3 included terms for model 2, diabetes, dyslipidemia and hypertension. No interaction was found between blood lead level and diabetes, dyslipidemia or hypertension.

Overweight and obese adults were defined based upon BMI measures of 25–29.9 kg/m^2^ and ≥30 kg/m^2^, respectively.
