# Supplementary material for: Blood lead level and its association with body mass index and obesity in China - Results from SPECT-China study
Source: Sci Rep. 2015 Dec 14;5:18299. doi: 10.1038/srep18299 (PMC4677308; doi:10.1038/srep18299)
Supplement: Supplementary Information [file srep18299-s1.pdf]

Blood lead level and its association with body mass index and obesity in China - Results from SPECT-China study

Ningjian Wang[1], Chi Chen[1], Xiaomin Nie[1], Bing Han[1], Qin Li[1], Yi Chen[1], Chunfang Zhu[1], Yingchao Chen[1], Fangzhen Xia[1], Zhen Cang[1], Meng Lu[1], Ying Meng[1], Hualing Zhai[1], Dongping Lin[1], Shiyong Cui[2], Michael D. Jensen[**3], Yingli Lu[1]

1 Institute and Department of Endocrinology and Metabolism, Shanghai Ninth People's Hospital, Shanghai JiaoTong University School of Medicine, Shanghai, China

2 The Center for Disease Prevention and Control of Baoshan District, Shanghai, China

3 Endocrine Research Unit, Mayo Clinic, Rochester, MN, USA

Supplementary Table S1 environmental and blood lead level by economic status

	Low economic status	High economic status
Lead in drinking water, ug/L	0.93 (0.36-1.62)	1.07 (0.50-2.42)
Lead in river water, ug/L	2.90 (1.85-4.83)	1.29 (0.34-2.99)*
Lead in rice, mg/kg	0.038 (0.028-0.094)	0.021 (0.012-0.030)*
Men		
N	616	1619
Age, yr	51 (45-60)	55 (43-63)*
Blood lead level, ug/L	39.0 (27.2-54.4)	46.9 (30.0-65.4)*
Body mass index, kg/m^2	24.5 (22.2-26.8)	24.2 (22.0-26.6)
Weight, %		
Overweight	40.4	35.3*
Obesity	5.5	4.9
Women		
N	992	2331
Age, yr	48 (40-60)	55 (44-62)*
Blood lead level, ug/L	34.1 (24.1-47.5)	39.9 (26.0-58.4)*
Body mass index, kg/m^2	23.2 (21.2-25.4)	23.7 (21.5-26.1)*
Weight, %		
Overweight	24.2	29.5*
Obesity	4.4	6.1*

* Denotes statistical significance at $P<0.05$ compared with low economic status.

Data were summarized as median (interquartile range) for continuous variables, or as number with proportion for categorical variables. Mann-Whitney U was used for continuous variables and Pearson chi-square test for categorical variables.

Overweight and obese adults were defined based upon BMI measures of 25-29.9 kg/m^2 and ≥30 kg/m^2, respectively.

Supplementary Table S2 environmental and blood lead level by rural/urban residence

	Rural residence	Urban residence
Lead in drinking water, ug/L	1.07 (0.39-2.02)	1.04 (0.54-2.59)
Lead in river water, ug/L	1.32 (0.50-2.99)	3.56 (2.38-5.11)*
Lead in rice, mg/kg	0.021 (0.010-0.031)	0.036 (0.020-0.078)*
Men		
N	1191	1044
Age, yr	58 (47-66)	50 (40-58)*
Blood lead level, ug/L	51.9 (35.4-71.0)	36.7 (24.8-52.3)*
Body mass index, kg/m^2	23.7 (21.5-26.2)	24.8 (22.7-27.1)*
Weight, %		
Overweight	32.4	41.6*
Obesity	4.0	6.3*
Women		
N	1913	1410
Age, yr	57 (46-64)	48 (39-58)*
Blood lead level, ug/L	43.0 (28.0-62.0)	33.0 (22.0-45.6)*
Body mass index, kg/m^2	23.8 (21.5-26.2)	23.2 (21.2-25.6)*
Weight, %		
Overweight	29.3	26.0*
Obesity	6.5	4.3*

* Denotes statistical significance at P<0.05 compared with rural residence.

Data were summarized as median (interquartile range) for continuous variables or as number with proportion for categorical variables. Mann-Whitney U was used for continuous variables and Pearson chi-square test for categorical variables.

Overweight and obese adults were defined based upon BMI measures of 25-29.9 kg/m^2 and ≥30 kg/m^2, respectively.

Supplementary Table S3 environmental and blood lead level by the territorial origin

	Jiangxi (low ES)		Zhejiang (high ES)	Shanghai (high ES)	
	Urban	Rural	Rural	Urban	Rural
**Men**					
N	465	151	586	579	454
Age, yr	49(43-55)	66(58-72)*	56(44-65)*	53(35-60)	58(48-64)*
BLL, ug/L	38.48(27.11-53.32)	43.00(28.00-56.77)	46.83(32.00-64.00)*	35.00(22.43-52.11)	61.08(46.56-83.32)*
BMI, kg/m2	25.1(22.7-27.1)	23.0(20.5-25.8)*	22.9(20.8-25.0)*	24.7(22.7-27.1)	25.3(23.0-27.3)
Weight, %					
Overweight	45.2	25.8*	23.4*	38.7*	46.3
Obesity	5.8	4.6	1.9*	6.7	6.6
**Women**					
N	713	279	786	697	848
Age, yr	44(39-52)	64(57-71)*	55(44-63)*	54(40-60)*	55(46-63)*
BLL, ug/L	33.96(24.20-45.80)	35.68(24.00-53.00)	34.00(24.00-50.36)	31.96(19.57-45.10)	53.32(38.52-76.26)*
BMI, kg/m2	23.0(21.1-25.3)	23.5(21.3-25.9)	22.7(20.6-25.1)	23.4(21.4-25.9)	24.8(22.5-27.4)*
Weight, %					
Overweight	24.1	24.4	22.6	28.0	37.1*
Obesity	4.2	5.0	3.8	4.4	9.6*

ES, economic status. * Denotes statistical significance at P<0.05 compared with urban in Jiangxi. Data were summarized as median (interquartile range) for continuous variables or as number with proportion for categorical variables. Kruskal-Wallis test were used for continuous variables and Pearson chi-square test for categorical variables. Overweight and obese adults were defined based upon BMI measures of 25-29.9 kg/m^2 and ≥30 kg/m^2, respectively.

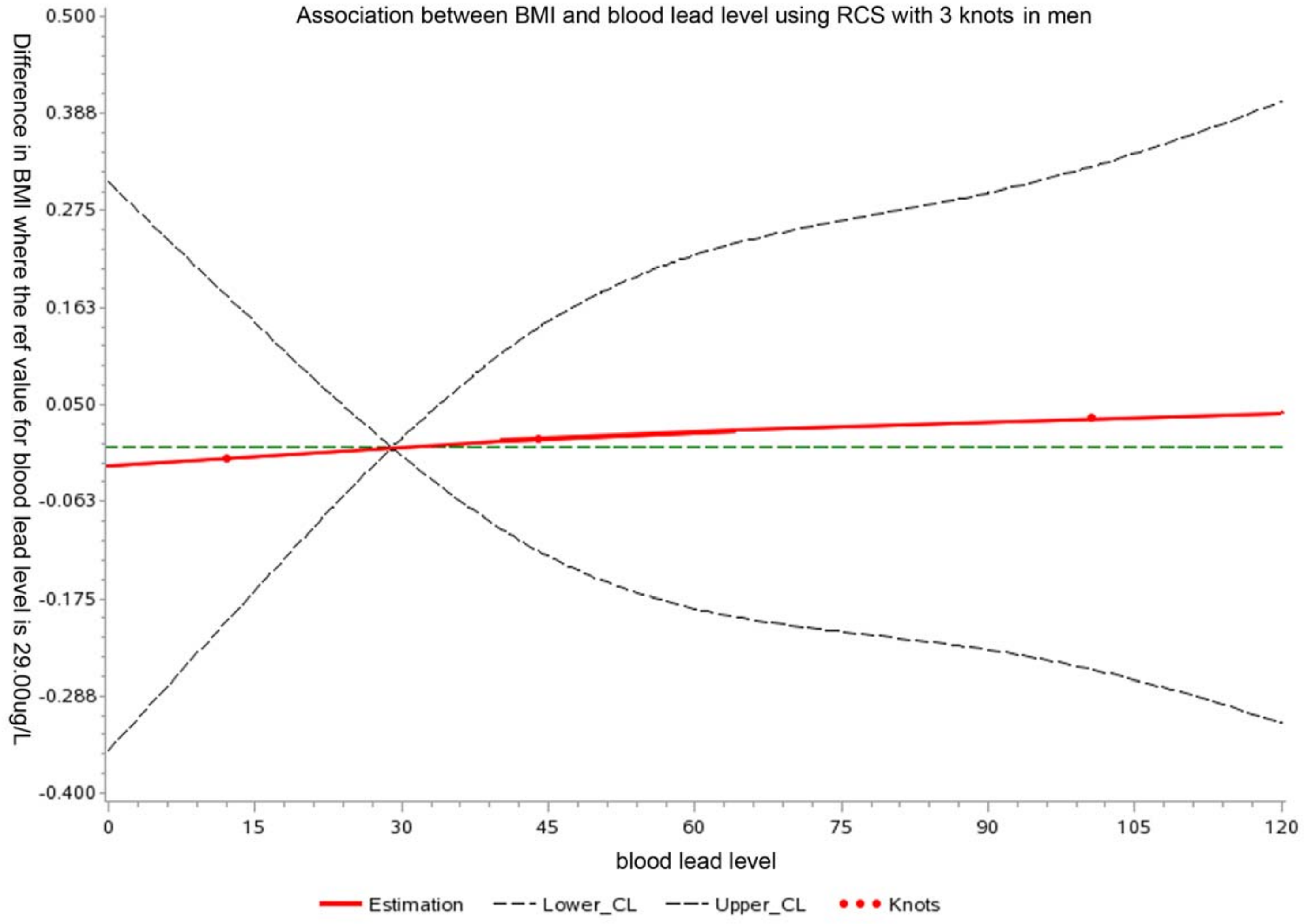

Supplementary Figure S1 Dose–response association between BMI and BLL in men adjusted for age, rural/urban residence, economic status, current smoking, diabetes, dyslipidemia and hypertension. A solid line shows dose–response curve between BLL and BMI. Y-axis represents the difference in BMI between individuals with any value of BLL with individuals with 29.00μg/L of BLL. The dashed lines represent the 95% confidence of interval. Knots are represented by dots.
